# Efficacy of Lovastatin on Learning and Memory Deficits Caused by Chronic Intermittent Hypoxia-Hypercapnia: Through Regulation of NR2B-Containing NMDA Receptor-ERK Pathway

**DOI:** 10.1371/journal.pone.0094278

**Published:** 2014-04-09

**Authors:** Xin-long Huo, Jing-jing Min, Cai-yu Pan, Cui-cui Zhao, Lu-lu Pan, Fei-fei Gui, Lu Jin, Xiao-tong Wang

**Affiliations:** The Center of Neurology and Rehabilitation, The Second Affiliated Hospital of Wenzhou Medical University, Wenzhou, China; Inserm U837, France

## Abstract

**Background:**

Chronic intermittent hypoxia-hypercapnia (CIHH) exposure leads to learnning and memory deficits in rats. Overactivation of N-methyl-D-aspartate receptors(NMDARs) can lead to the death of neurons through a process termed excitotoxicity, which is involved in CIHH-induced cognitive deficits. Excessively activated NR2B (GluN2B)-containing NMDARs was reported as the main cause of excitotoxicity. The ERK1/2 (extracellular signal-regulated kinase 1/2) signaling cascade acts as a key component in NMDARs-dependent neuronal plasticity and survival. Ca2+/calmodulin-dependent protein kinase II (CaMKII), synapse-associated protein 102 (SAP102) and Ras GTPase-activating protein (SynGAP) have been shown to be involved in the regulation of NMDAR-ERK signalling cascade. Recent studies revealed statins (the HMG-CoA reductase inhibitor) have effect on the expression of NMDARs. The present study intends to explore the potential effect of lovastatin on CIHH-induced cognitive deficits and the NR2B-ERK signaling pathway.

**Methods and Findings:**

Eighty male Sprague Dawley rats were randomly divided into five groups. Except for those in the control group, the rats were exposed to chronic intermittent hypoxia-hypercapnia (CIHH) (9∼11%O_2_, 5.5∼6.5%CO_2_) for 4 weeks. After lovastatin administration, the rats performed better in the Morris water maze test. Electron microscopy showed alleviated hippocampal neuronal synaptic damage. Further observation suggested that either lovastatin or ifenprodil (a selective NR2B antagonist) administration similarly downregulated NR2B subunit expression leading to a suppression of CaMKII/SAP102/SynGAP signaling cascade, which in turn enhanced the phosphorylation of ERK1/2. The phosphorylated ERK1/2 induced signaling cascade involving cAMP-response element-binding protein (CREB) phosphorylation and brain-derived neurotrophic factor (BDNF) activation, which is responsible for neuroprotection.

**Conclusions:**

These findings suggest that the ameliorative cognitive deficits caused by lovastatin are due to the downregulation of excessive NR2B expression accompanied by increased expression of ERK signaling cascade. The effect of NR2B in upregulating pERK1/2 maybe due, at least in part, to inactivation of CaMKII/SAP102/SynGAP signaling cascade.

## Introduction

Chronic obstructive pulmonary disease (COPD) is a major cause of chronic morbidity and mortality [1]. Many reports reveal its harmful effect on learning and memory function, which cannot be explained just by coincidence or by depression [2]. Autopsy reports and animal experiments confirm that this cognitive disorder is related to pathological changes in the central nervous system (CNS), particularly in the hippocampus [3], [4]. Chronic intermittent hypoxia hypercapnia (CIHH) is an animal model similar to pathophysiological process in patients with COPD [5]. In our previous study, we confirmed that the exposure of CIHH deteriorated the learning and memory ability of the experimental rats as the exposure time was prolonged after two weeks [6], [7].

Glutamate is one of the most important excitatory neurotransmitters in CNS, playing a vital role in mediating synaptic plasticity and receptor function in the regulation of learning and memory [8]. NMDARs are cation channels that are gated by the neurotransmitter glutamate. Normally, NMDARs are essential regulators of synaptic plasticity, neuronal development and synaptic transmission [9], [10]. However, in neurodegeneration and hypoxic stress, excessive release of glutamate leads to sustained activation of NMDA-type glutamate receptors and downstream effectors, resulting in neuronal damage and cell death. This process is known as synaptic excitotoxicity [11]. Synaptosome excitotoxicity is considered a pivotal factor causing CNS damage [12], [13].

NMDARs are tetrameric protein complexes composed of NR1 subunits and at least one type of NR2 subunit(NR2A-D, also known as GluN2A-D). NR2A and NR2B are the predominant NR2 subunits in the neurons of the hippocampus and prefrontal cortex. However, the NR2A and NR2B show difference in synaptic localization and functional properties. The activation of synaptic NR2A is involved in neuroprotection while the stimulation of extrasynaptic NMDARs, which are predominantly composed of NR2B subunits [14], play an important role in learning and memory. However, in pathophysiological conditions, such as ischaemia and/or hypoxia, or neurodegeneration,NR2B subunits trigger cell destructive pathways and the persistent activation of extrasynaptic NR2B is responsible for excitotoxicity [15], [16]. The mitogen-activated protein kinase phosphatase (MAPK) signal transduction system, the eventual common pathway in eukaryotic cells for the transmission of various information, transduces extracellular information to the nucleus and regulates cell growth and differentiation [17], [18]. One of the best-characterized MAPK pathways involves the 44-and 42-kDa isoforms, which are recognized as extracellular signal-regulated kinase 1 and 2 (ERK1/2). Studies have demonstrated ERK signalling cascade is a crucial pathway in mediating NMDAR-dependent neuronal plasticity and survival [19], [20]. Normal expressed NMDAR-dependent signalling activates the ERK1/2 cascade with pro-survival consequences including CREB and BDNF activation [21], pro-apoptotic factors inactivation, and antagonizing GSK3β-induced apoptosis [22]. Other researches found that the ERK cascade is closely linked to the NMDAR after they observed the direct interaction of ERK with NR2B subunit and calcium-calmodulin-regulated guanine-nucleotide exchange factor RasGRF1 [23]. However, how NR2B regulates the ERK activity remains obscure.

NMDARs are localized at the postsynaptic membrane of neurons. The NMDA receptor-interacting proteins, including PSD95, PSD93 and synapse-associated protein 102 (SAP102) are concentrated in the postsynaptic density (PSD), which composed of membrane-associated guanylate kinases (MAGUKs) [24]. The MAGUKs consist of three PDZ domains, a Src homology 3 (SH3) domain and an inactive guanylate kinase (GK) domain [25]. As a PSD-associated guanylate kinase, SAP102 demonstrates the strongest interaction with NR2B to regulate the clustering and anchoring of NR2B in the synaptic membrane [26]. Previous studies also discovered that the N terminus of SAP102 contains a NR2B-specific NMDAR binding site [27]. Normally, CaMKII is a large holoenzyme and activated by calcium influx to the synapse (mainly via NMDARs) [28]. The activated CaMKII can bind to NR2B with high affinity, whereas CaMKII binds to NR2A with low affinity and the association between active CaMKII and NR2B is required for the maintenance of synaptic plasticity [29]. Under hypoxic stress, the sustained increasing concentration of extracellular glutamate induces the excessive activation of NMDARs, resulting in uncontrolled Ca2+ influx and activation of CaMKII and its downstream effectors, which triggers excitotoxicity. SynGAP is a synaptic Ras GTPase-activating protein (also named RasGAP) and highly concentrates at excitatory synapses in hippocampal neuron. SynGAP is associated with a large complex with PSD95/SAP90, SAP102 and the NMDARS. SynGAP is activated by Ca2+/calmodulin-dependent protein kinase II (CaMKII) and suppresses the Ras-extracellular signal-regulated kinase (ERK) pathway via stimulating GTPase activity of Ras [30], [31].

Excitotoxicity has been investigated as a novel target for neuroprotective strategy because of its essential role in acute and neurodegenerative disorders. NMDARs antagonists are expected to display these neuroprotective effects. However, a number of clinical trials using NMDAR antagonists have failed because of their intolerable side effects or lack of medical efficacy. But a series of recent studies demonstrated statins, the HMG-CoA reductase inhibitor, could downregulate the expression of NMDARs and relieve the excitotoxicity [32], [33], [34]. This finding has cast a new light on excitotoxicity-induced cell death and neuronal neurodegeneration. Lovastatin, a classic cholesterol-lowering drug, is widely used in various illnesses for its multiple efficient effects including anti-inflammation, antioxidation, anti-apoptosis and anticoagulation [35], [36]. The question remains whether lovastatin also has a protective effect on NMDARs to relieve CIHH-induced excitotoxicity and the underlying regulatory mechanism. Therefore the CIHH rat model is applied to study if and how lovastatin regulates the NR2B subunit. The NR2B-specific antagonist ifenprodil is used here as the positive control.

## Materials and Methods

### Ethic Statement

All animal procedures were approved by the Ethics Committee of Wenzhou Medical University (Approved No. wydy2013–0028). All efforts were made to reduce the number of animals and to minimize their suffering in accordance with the ethical guidelines for animal research.

### Animals and drug treatment

Male Sprague-Dawley rats weighting 200±10 g at the beginning of the experiments were purchased from the Laboratory Animal Center of Wenzhou Medical University. Rats were housed under specific pathogen-free (SPF) conditions with a 12/12 h dark/light cycle in 23±1°C room temperature. Rats had free access to food and water. Rats were randomly categorized into five groups (16 per group): (i) normal control group (NC); (ii) hypoxia-hypercapnia group (HH); (iii) hypoxia-hypercapnia + DMSO and PBS group (HS); (iv) hypoxia-hypercapnia + lovastatin group (HL) and (v) hypoxia-hypercapnia+ ifenprodil group (HI). The CIHH model was performed as previous described [6], [7]. The rats in the latter 4 groups were intermittently placed in a closed chamber that was ventilated with an elevated CO_2_ gas mixture (9%–11% O_2_+6.5%–7.5%CO_2_ in N_2_). The exposure cycle repeated every 4 minutes for 8 h/day (from 8am to 4pm), 7 days/week for 4 weeks. Lovastatin (Sigma-Aldrich, St. Louis, MO, USA) at a dose of 2 mg/kg or DMSO and PBS at the same dose or 5 mg/kg ifenprodil (Sigma-Aldrich, St. Louis, MO, USA) was intraperitoneal injected into the rat, once a day after the exposure cycle was completed for 4 weeks. The solvent of lovastatin or ifenprodil was DMSO and PBS. We choosed the dose of 2 mg statin/kg feed equates to 20 mg/kg body weight of clinical dose of statins. The chamber where the NC group placed was flushed with room air instead of N_2_.

### Morris water maze

The Morris water maze was performed as previous described by Morris [37]. The Morris water maze system consisted of a white circular pool, 1.7 m in diameter and 40 cm in height, filled with 35 cm black dyed water (22±1°C). The maze was artificially divided into four imaginary quadrants, and a hidden 10 cm diameter platform was positioned 1 cm below the water surface in one quadrant. Several cues visible to the rats were fixed on the walls of the maze. The task consisted of two training days and three test days. In the task, the rat was placed in every quadrant sequentially, facing the wall. Immediately, the latency of the rat to find the platform and the swimming paths were recorded by a video camera suspended above the maze, which interfaced with an image analyzer (HVS System, Buckingham, UK). A maximum of 60 s were allowed for the rat to find the platform. After 60 s, the rat was guided to the platform, and the latent period was recorded as 60 s. Thirty seconds of rest on the platform were allowed before the experiment was initiated in the next quadrant. The training was ended when four trials were completed on that day. On the last day, the platform was removed before the test. The times the animal crossed the prior location of the platform were recorded. Individual trials were averaged by blocks of four trials conducted on the same day.

### Ultrastructure observation of the hippocampus with electron microscopy

After the Morris water maze, two rats were randomly selected from each group for electron microscopy. The rats were killed with an overdose of 7% chloral hydrate and then perfused transcardially with saline (NaCl 0.9%) followed by 4% paraformaldehyde in 0.1 M phosphate buffer, pH 7.4 (Sigma, USA). The hippocampal CA1 region were removed rapidly. The electron microscopy specimens were processed in a conventional way, fixed in 2.5% glutaraldehyde and post-fixed in 1% osmium tetroxide (Sigma, USA) at room temperature. The specimens were embedded with Epon812. Coronal ultra-thin sections were cut using a LKB2088 vibratome and placed on single-hole grids. The ultra-thin sections (80 nm) were stained with 2% lead citrate (Merck, Germany) and 1% uranyl acetate (Merck, Germany) and the ultrastructure changes of pyramidal cells were examined using a transmission electron microscope (Hitachi H-7500 electron, Japan).

### Isolation of the Synaptosome from Rat Hippocampus

After 4 weeks CIHH exposure, 10 rats in each group were deeply anesthetized with 7% chloral hydrate i.p. and and transcardially perfused with saline (NaCl 0.9%). The hippocampus were isolated and we isolated the synaptosome as follows. First, the hippocampus were combined and homogenized in solution A (0.32 M sucrose ten times the tissue volume, 1 mM MgCl_2_, 0.5 mM CaCl_2_ and NaHCO_3_ containing protease inhibitor cocktail) at 4°C using a hand-held disperser. The resultant homogenates was centrifuged at low speed (1400 g for 10 min) to discard the sediment containing the nucleus. The supernatants were obtained and centrifuged at 17000 g for 20 min. After this step, the resulting pellet containing synaptosomes was obtained. The pellet was suspended in solution B (0.32 M sucrose and 1 mM NaHCO_3_ containing protease inhibitor cocktail). Next, a sucrose density gradient was applied to extract the pure synaptosome. The suspension liquid was added to the sucrose gradients (composed of 0.8 and 1.2 M sucrose) and then centrifuged in a SW41 Ti Beckman rotor at 54,000 g for 2 h. Synaptosome suspensions at the interface between 0.8 and 1.2 M sucrose were collected. The collected suspensions were diluted with solution B and centrifuged for 15 min at 20000 g. Precipitates were the synaptosomes. All the centrifugation processes were at 4°C, and the proteins obtained were stored at −80°C.

### Western blotting analysis

Protein concentration was determined by the BCA protein assay kit. Proteins (20 μg) were resolved in SDS-polyacrylamide gels and electrotransferred onto polyvinylidene difluoride (PVDF) membranes (Millipore, Billerica, MA). Nonspecific binding sites on the blotting membrane were blocked with 5% non-fat dry milk for 2 h and incubated at 4°C overnight with mouse anti-NR2B (1∶1000, Abcam), rabbit anti-SAP102 (1∶500, Santa Cruz), rabbit anti-CaMKII (1∶1000, Cell Signaling), rabbit anti-SynGAP (1∶1000, Abcam), rabbit anti-pERK1/2 (1∶2000, Cell Signaling), rabbit anti-pCREB (1∶1000, Cell Signaling Technology) and rabbit anti-BDNF (1∶1000, Sigma). The proteins were detected with anti-rabbit or anti-mouse secondary antibody for 2 h at room temperature. Then, the proteins were visualized with BeyoECL Plus reagents and captured by an enhanced chemiluminescence system (ECL kit, Pierce Biotechnology, Inc.).

### Double Immunofluorescence Labeling

Four rats from each group were sacrificed after the Morris water maze test. The rats were deeply anesthetized by intraperitoneal injection of 7% chloral hydrate and transcardially perfused with saline (NaCl 0.9%). The hippocampus were removed rapidly and dehydrated in 0.1 M phosphate buffer containing 30% sucrose until sinking to the bottom. Hippocampus were sectioned coronally at 30 μm thickness using a freezing microtome. Next, the hippocampal CA1 sections were used for double-labeling immunofluorescence. Sections were washed for 3 hours in PBS containing 0.1% Triton-X 100 (PBS-Tx) and immersed for 1 hour in a solution of 5% normal goat serum (Vector) in PBSTx. Samples were incubated overnight with a mouse monoclonal antibody to NR2B (diluted to 1∶100 in PBS) and a rat polyclonal antibody to pERK1/2 (diluted to 1∶500 in PBS). Samples were washed three times with PBS. Then, they were incubated for 1 hour at room temperature in a 1∶200 dilution of Cy3-conjugated goat anti-rabbit IgG (from Chemicon) and fluorescein isothiocyanate (FITC)-conjugated goat anti-mouse IgG (from Chemicon). The sections were mounted and examined using an Olympus FluoView FV500 confocal microscope. Control sections incubated with PBS instead of primary antibodies.

### Statistical analyses

The Morris water maze cumulative distance and latency were analyzed using repeated measures ANOVA (RM ANOVA) with group as between-subjects factors and day of trail as within-subjects factors. The date of water maze probe trial and western blotting assays were performed by one-way ANOVA followed by a post hoc comparison test using LSD (equal variances assumed) or Dunnett's T3 (equal variances not assumed) test to confirm the significant difference between the groups. p<0.05 was considered to be statistical significant. The data were expressed as the means ± SD.

## Results

### 1. Lovastatin treatment improves spatial learning and memory ability in CIHH exposure rats

**Figure 1:**
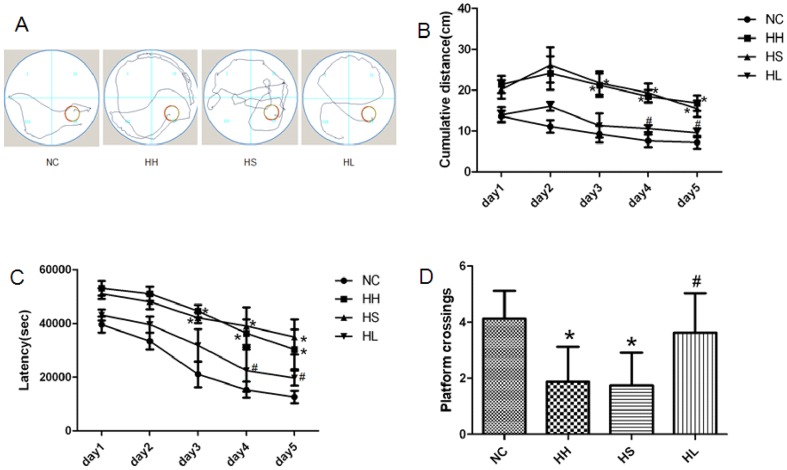
Performance of the rats in the Morris water maze. (A) The pathway taken to search for the hidden platform. (B) Cumulative distance to reach the platform. (C) Latency to find the platform. (D) Number of platform crossings. Values are expressed as mean ± SD. * p<0.05 when compared to the NC group, and # p<0.05 when compared to the HH group. NC = normal control group; HH = hypoxia-hypercapnia group; HS =  hypoxia-hypercapnia+DMSO and PBS group; HL = hypoxia-hypercapnia+lovastatin.

The pathway (Figure 1A) of rat exploring the hidden platform in the target quadrant was recorded. In the previous two days, each group demonstrated no significant difference in the cumulative distance and latency. From the third day on, the cumulative distance (Fig. 1B) and latency (Fig. 1C) increased significantly (p<0.05) in the HH and HS groups when compared to the NC group. But the group with lovastatin administration showed significantly (p<0.05) decrease in the cumulative distance (Fig. 1B) and latency (Fig. 1C) relative to HH group for the latter two days of the acquisition trial. The probe trial test was applied after five days of training, and the result demonstrated that CIHH exposure led to a significantly (p<0.05) decrease in the number of platform crossings (Fig. 1D) compared to the NC group. Conversely, lovastatin increased (p<0.001) the number of platform crossings (Fig. 1D).

### 2. Lovastatin treatment ameliorates hippocampal synapse damage in CIHH exposure rats

**Figure 2:**
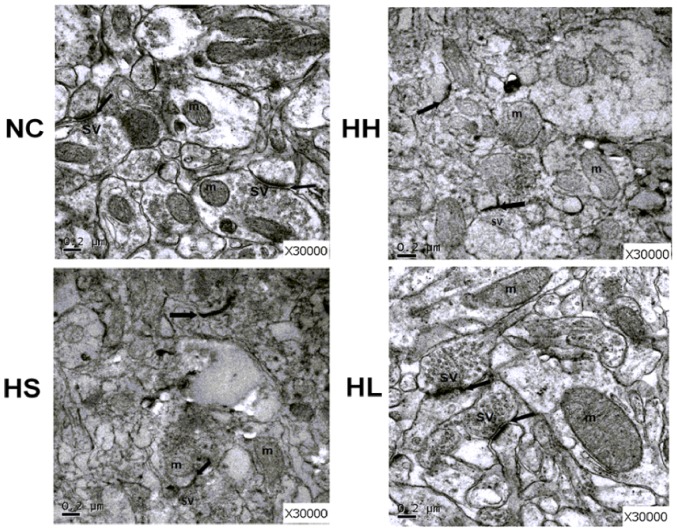
Ultrastructure changes in the hippocampus of each groups. The picture shows the ultrastructure changes of pyramidal cells in the hippocampal CA1 region in each group viewed by electron microscopy. Scale bars indicate 0.2 μm. The black arrow points to the synaptic cleft. Synaptic vesicles (sv) and mitochondria (m) are also marked in Figure 2. NC = normal control group; HH = hypoxia-hypercapnia group; HS =  hypoxia-hypercapnia+DMSO and PBS group; HL = hypoxia-hypercapnia+lovastatin.

As hippocampus is the most important region for learning and memory, we used transmission electron microscopy (TEM) to observe the pathologic damage in the hippocampus CA1 area (Fig. 2). In the NC group, the structures of presynaptic and postsynaptic membrane and the mitochondria cristae were clear. A large pool of round synaptic vesicles collected in the presynaptic terminals, with a thick opposing postsynaptic density. In the HH and HS groups, synaptic edema emerged in dendritic spines and axons. This was coupled with obscure boundaries and the disappearance of the synaptic vesicles and postsynaptic density. The synaptic cleft narrowed as a result of edema. Compared with the HH group, the HL group demonstrated amelioration of synaptic edema, increased the width of the synaptic cleft, had intact synaptic membranes and improved postsynaptic density.

### 3. Lovastatin or ifenprodil treatment downregulates NR2B expression and upregulates pERK1/2 expression

**Figure 3:**
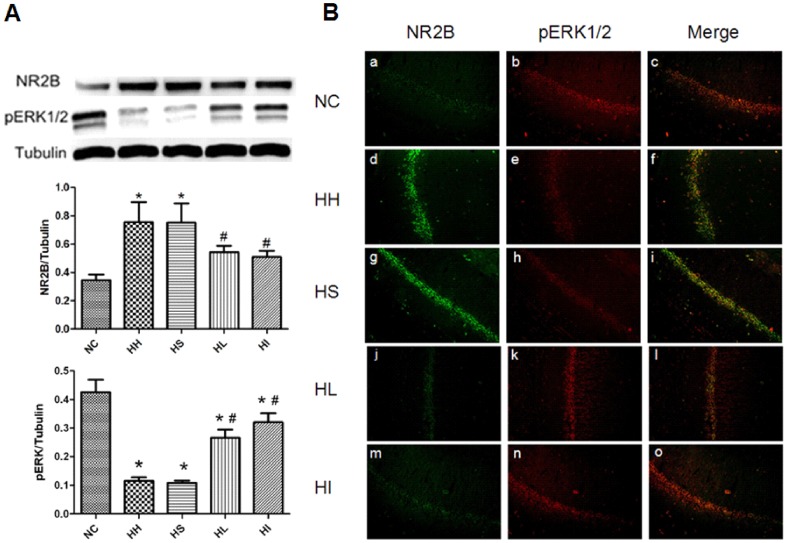
Lovastatin or ifenprodil administration reduces NR2B subunit and upregulates pERK expression. Bilateral hippocampus from each group were collected for western blotting and double immunofluorescence staining. (A) Western blotting shows NR2B and pERK levels in the hippocampus in the five groups. Tubulin was used as a loading control. The optical density values were normalised to their respective Tubulin loading control, and the means±SD were graphed (relative expression) to semiquantitatively compare the protein levels. *p<0.05 vs. NC group, #p<0.05 vs. HH group; (B)The double immunofluorescence staining observed by confocal microscope shows the colocalization for NR2B (green fluorescence) and pERK (red fluorescence) in the pyramidal cells of hippocampal CA1 region. Original magnification×200. NC = normal control group; HH = hypoxia-hypercapnia group; HS =  hypoxia-hypercapnia+DMSO and PBS group; HL = hypoxia-hypercapnia+lovastatin; HI =  hypoxia-hypercapnia +ifenprodil group.

As NR2B was reported to be responsible for excitotoxicity, we first investigated NR2B subunit by western blotting. The expression of NR2B was drastically elevated (p<0.05) in the HH and HS groups, but downregulated (p<0.05) by lovastatin administration(Fig. 3A). To further confirm the negative role of NR2B in our model, we introduced ERK signalling cascade, which is a crucial pathway in mediating NMDAR-dependent neuronal plasticity and survival. After CIHH exposure, the phosphorylated ERK1/2 (Fig. 3A) was decreased(p<0.05) but significantly(p<0.05) elevated with lovastatin administration. To determine the relationship between NR2B and pERK1/2, the NR2B subunit antagonist ifenprodil was applied as a positive control. The results showed the phosphorylation of ERK1/2 was influenced by NR2B. We further investigated NR2B subunit and pERK1/2 expression of pyramidal cells in the hippocampal CA1 region by double immunofluorescence staining (Fig. 3B). The co-localization of NR2B with pERK in CA1 manifested a relationship consistent with the results of the western blottings. Both lovastatin and ifenprodil administration could downregulate NR2B subunit expression and thus elevating pERK1/2 when compared to the HH group.

### 4. Lovastatin or ifenprodil administration promotes BDNF and pCREB activation through ERK1/2 phosphorylation

**Figure 4:**
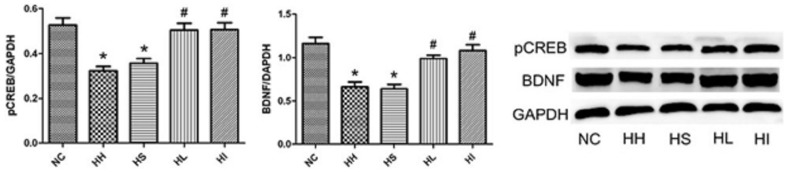
Lovastatin or ifenprodil administration promotes expression of BDNF and pCREB. Bilateral hippocampus from each group were collected for western blotting analysis. GAPDH was used as a loading control. The optical density values were normalised to their respective GAPDH loading control, and the means±SD were graphed (relative expression) to semiquantitatively compare the protein levels. *p<0.05 vs. NC group, #p<0.001 vs. HH group. NC = normal control group; HH = hypoxia-hypercapnia group; HS =  hypoxia-hypercapnia+DMSO and PBS group; HL = hypoxia-hypercapnia+lovastatin; HI =  hypoxia-hypercapnia +ifenprodil group.

To identify downstream effects of ERK1/2 phosphorylation, we measured the ERK1/2 downstream targets phospho-CREB and BDNF. BDNF was shown to be a critical neurotrophin to promote neuronal survival by coupling of ERK1/2/CREB to pCREB-mediated transcription. Four weeks of CIHH exposure resulted in a significant decrease of BDNF and pCREB when compared to the NC group (Fig. 4) (p<0.05), However, lovastatin or ifenprodil prevented this reduction relative to the HH group (p<0.05).

### 5. Lovastatin or ifenprodil reduces the expression of CaMKII, SAP102 and SynGAP

**Figure 5:**
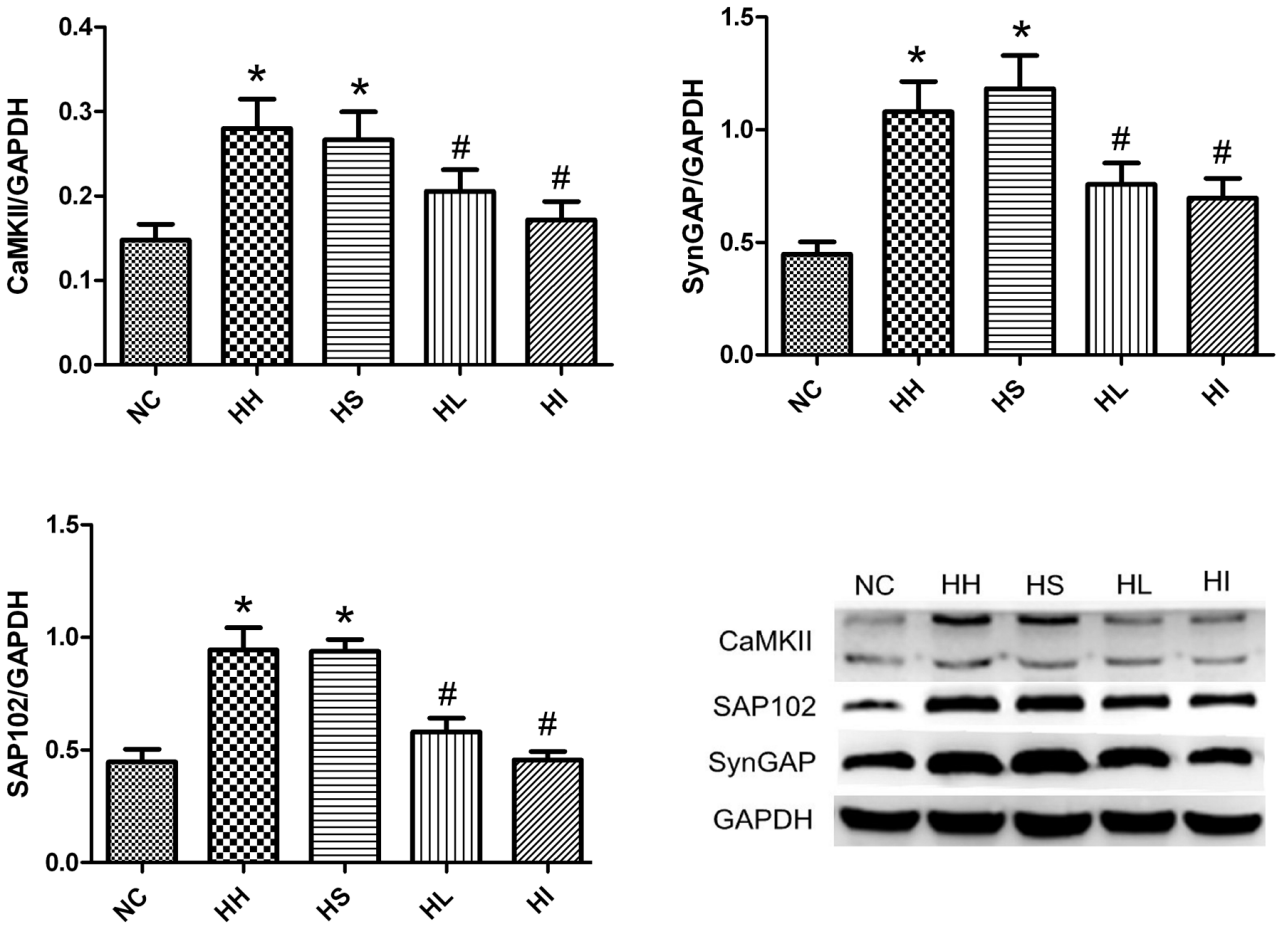
Inhibitory effects of lovastatin or ifenprodil on the CIHH-induced expression of CaMKII, SAP102 and SynGAP. Hippocampal homogenates were isolated from the brains of five groups of rats. Analyses of the expression of CaMKII, SAP102 and SynGAP were performed by western blotting. GAPDH was used as a loading control. The optical density values were normalised to their respective GAPDH loading control, and the means±SD were graphed (relative expression) to semiquantitatively compare the protein levels. *p<0.05 vs. NC group, #p<0.05 vs. HH group. NC = normal control group; HH = hypoxia-hypercapnia group; HS =  hypoxia-hypercapnia+DMSO and PBS group; HL = hypoxia-hypercapnia+lovastatin; HI =  hypoxia-hypercapnia +ifenprodil group.

CaMKII, SAP102 and SynGAP have been shown to be involved in the regulation of NMDAR-ERK signalling cascade with the unclear controlled mechanism. As most evidence was obtained from cell-based experiments, we studied the process *in vivo*. Four-week CIHH exposure significantly (p<0.05) increased the expression of CaMKII, SAP102 and SynGAP accompanied with downregulated pERK1/2 in the HH and HS groups compared to the NC group (Fig.5) (p<0.05). In contrast, four-week CIHH exposure with lovastatin or ifenprodil administration resulted in a significant (p<0.05) reduction in those proteins and coupled with increased pERK1/2 compared to the HH group (p<0.05).

## Discussion

Cognitive impairment in COPD has been widely acknowledged, but the exact pathogenesis is controversial and the treatment options remain inconclusive [38]. Studies implicated that in hypoxic stress, excessive and/or prolonged activation of NMDARs, followed by intracellular calcium (Ca^2+^) overload, lead to excitotoxicity and cell death [39], [40]. In our present study, chronic lovastatin administration improved the performance of CIHH rats in the Morris water maze and ameliorated their spatial learning and memory ability. Further observation corroborated that chronic administration of lovastatin inactivated NR2B subunit coupled with increased pERK1/2 expression and its downstream target including pCREB and BDNF in the hippocampal area. Similarly, the effect was confirmed by the administration of selective NR2B antagonist ifenprodil. An increase in CaMKII, SAP102 and SynGAP was observed in CIHH-induced cognitive impairment and these effects were reversed by lovastatin or ifenprodil. Our findings suggested that the novel neuroprotective effects of lovastatin against CIHH induced excitotoxicity and neuron damage occurred through downregulating NR2B and activating the ERK signaling cascade, which are at least partly regulated by CaMKII/SAP102/SynGAP signaling cascades.

Although COPD has long been recognized as a respiratory disease due to the irreversible loss of lung function, its systemic effects are attracting increasing attention [41], [42]. In particular, the learning and memory deficits caused by COPD becomes a disabling problem [43]. Chronic exposure to hypoxia leads to changes of various factors including glutamate excitotoxicity, depletion of growth factors, alteration in cellular biochemistry, etc, and the hippocampus is reported to be the most vulnerable region [44], [45]. Statins, a common treatment for hypercholesterolemia, have been shown to exert beneficial effects in various diseases. Epidemiologic studies have shown that taking statins can not only significantly reduce cholesterol, but can also prevent and slow down the occurrence and progress of stroke and dementia [46], [47]. However, the application of statins in treating COPD-related cognitive impairment is still unknown. In this study, the administration of lovastatin in CIHH rats leads to improved learning and memory, manifested by the better performance in both the acquisition test and probe trials of the Morris water maze. Furthermore, lovastatin shows a protective role in alleviating synaptic edema and maintaining the synaptic stabilization.

The glutamate mediated NMDARs play a key role in neuronal plasticity, learning and memory. However, inappropriate activation or overstimulation of the NMDARs trigger excitotoxicity and result in neuronal death or degeneration in different disorders, including hypoxc and/or ischaemic brain injury and chronic neurodegenerative diseases [48]. Recently, the molecular structure of NMDA receptors has been further understood and these findings provide the foundation for designing subtypeselective antagonists. NR2A and NR2B are the dominating subunits of the four NMDA NR2 subunits in the CNS and play different roles in synaptic plasticity. NR2A-containing NMDA receptors are located primarily at synaptic sites and mediate neuroprotection, whereas NR2B-containing receptors are predominantly outside synapses and play indispensable roles in neurodegenerative and apoptotic pathways [49]. Further studies regard the selective activation of extrasynaptic NR2B subunits as the trigger of excitotoxicity [50]. In an ischemic brain injury model, the application of the selective NR2B antagonist ifenprodil significantly attenuated the ischemia-induced brain damage and behavioral changes [51]. Hydroxysafflor yellow A, a traditional Chinese medicine extract, alleviates excitotoxic neuronal death by down-regulation of NR2B [52]. Therefore, the negative control of NR2B subunit has been suggested as a novel target to reduce excitotoxicity.

In this study, we observed lovastatin downregulates NR2B subunit expression under CIHH condition, but the underlying mechanism is worth thinking over. Although the central nervous system (CNS) represents only 2% of the human body weight, it accounts for almost 25% of the total cholesterol. Therefore, an increasing number of studies have emphasized the relationship between cholesterol and neurons [53]. The cholesterol-rich detergent-resistant membrane domains (DRMs) are preferentially considered. DRMs, commonly referred to as lipid rafts, are actually cell signaling platforms in plasma membranes. DRMs selectively recruit specific proteins as a function of cell status or in response to different treatments [54]. It has been reported previously that 50% of NMDA receptors are associated to lipid rafts [55]. Cholesterol depletion is reported to lead to an inhibition of calcium entry induced by NMDA, AMPA or kainate application, inferring that lipid rafts contribute to the regulation of ionotropic glutamate receptor function [56]. Our study demonstrates that lovastatin downregulates membrane expression of the NR2B subunit; a novel suggestion is that lovastatin may induce a dissociation of NR2B from the lipid raft fraction by reducing membrane cholesterol on the functionality of NR2B-NMDA receptors. The cholesterol depletion by lovastatin leads to an inhibition of calcium entry induced by NR2B-NMDA receptors, thus reducing the excitotoxicity caused by glutamate accumulation. Meanwhile, after four weeks administration with a selective antagonist ifenprodil, the result of decreased NR2B expression seems controversial to some previous studies reporting a prolonged blockade of the receptor promoted the expression of the target receptors [57], [58]. Since recent studies have found the effect of ifenprodil had a slow onset and the blockade was relatively modest [59], we speculate the low-dose administration of a modest antagonist might mainly lead to a promotion of sensitivity rather than upregulation of the receptors,at least in our model. In addition, the apoptotic-related neuronal death might also contribute to the decline of the overall quality of the NR2B (data not show here). Further study will be conducted to prove the conjecture.

As extrasynaptic NR2B is the main factor that triggers excitotoxicity, then how is it that synaptic NR2B transfers to the extrasynapse? SAP102, as an important member of MAGUKs family, controls synapse formation by clustering neurotransmitter receptors in axons and dendrites of matured neurons [60]. Studies have shown that SAP102 localizes closely with NR2B by PDZ domin [27]. Recent findings have also identified a secondary non-PDZ NR2B binding site in the N-terminal domain of SAP102, which might allow for preferential binding of SAP102 and NR2B [27]. In present studies, SAP102 significantly downregulated after lovastatin or ifenprodil administration accompained by decreasing NR2B. As SAP102 is a high mobile factor, we conjectured that the close interaction between NR2B and SAP102 is vital for trafficking of NR2B-containing NMDARs out of the synapse. However, further studies still need to exclude whether other factors also regulate the removal of NR2B.

To better evaluate the putative therapeutic role of lovastatin in preventing extrasynaptic NR2B-induced excitotoxicity, the mechanism underlying this protection was investigated. Previously, scholars have perceived that ERK activation occurs as a calcium-dependent NMDA receptor-mediated response, transferring extracellular stimuli to the nucleus and controlling the synaptic plasticity and learning. The phosphorylation of CREB, as a downstream target of ERKs, is also a necessary component for learning and memory formation processes [61]. The brain-derived neurotrophic factor (BDNF), one of the target genes of CREB, enhances cell survival and modulates synaptic activity [62]. Downregulation of BDNF could occur through a suppression of its transcription factor phospho-CREB [62], which also confirmed in our rat model and reversed by lovastatin or ifenprodil. ERK1/2 activation is thought to be an crucial defense mechanism against hypoxia/ischemia, which is essential for the inhibition of apoptosis and promotion of neurotrophin [63]. When the mouse's cortical neurons were exposed to cytotoxic hypoxia, ERK1/2 was activated, blocking increased hypoxia-induced cell death [63]. However, in our rat model, pERK1/2 expression was downregulated, which contradicted with the traditional view as to the active pERK1/2 promoting neuron survival with hypoxia. This phenomenon may be due to the fact that in our chronic hypoxia model, excitotoxicity has been already trigged, excessive pERK1/2 activation will only result in more energy consumption by neurons and exacerbate neuron survival. We conjecture some spontaneous protective mechnisms may be activated to inhibit this process.

In the present study, the upregulated NR2B and the downregulation of pERK1/2 were first observed after CIHH exposure. But the lovastatin administration decreased NR2B subnits followed by increased pERK expression. To confirm whether NR2B regulating the pERK, a selective NR2B antagonist ifenprodil was introduced and its application indeed drastically inhibited NR2B expression and led to the promotion of ERK phosphorylation. This data was confirmed by western blotting and double immunofluorescence staining. However, the question remains how the upstream NR2B regulates the ERK expression which is located far downstream. Studies have discovered that after NMDA stimulation, the increased CaMKII directly stimulate SynGAP, a Ras GTPase-activating protein, thereby inhibiting the ERK pathway [30], [31]. SynGAP also associates with NMDARs through binding to MAGUKs family proteins such as SAP102 [64]. In this study, after lovastatin or ifenprodil administration, decreased NR2B, CaMKII and SynGAP were found, resulting in the ERK cascade signaling activation as indicated by the increased pERK1/2, pCREB and BDNF expression. The disruption of overactivated NR2B and postsynaptic proteins and the upregulated expression ERK signaling cascade in the hippocampus may explain the neurobehavioral protection by lovastatin administration.

To summarize, this study illustrates the potential of lovastatin as a long-term, low risk therapy for CIHH-induced learning and memory deficits. More importantly, the possible protective mechanism is in the downregulation of NR2B subunit expression. NR2B inhibition negatively regulates the CaMKII/SAP102/SynGAP signaling pathway and stimulates the pro-survival phosphorylation of ERK and its downstream effectors. This mechanism plays an important role in alleviating hypoxia-hypercapnia-induced injury. To our best knowledge, this is the first time that a study has demonstrated that statins can downregulate the NR2B subunit in hypoxia-hypercapnia stress and may shed light on the treatment of COPD-induced cognitive impairment.
